# Diet and the Risk of Multiple Sclerosis: Evidence With UK Biobank Nested Case–Control Study and Mendelian Randomization Analysis

**DOI:** 10.1002/mnfr.70313

**Published:** 2025-11-21

**Authors:** Lian Chen, Xiao‐Wei Pang, Luo‐Qi Zhou, Wen‐Hui Song, Lu‐Yang Zhang, Li‐Fang Zhu, Wan‐Ning Li, Ming‐Hao Dong, Sheng Yang, Jun Xiao, Shuo‐Qi Zhang, Wei Wang, Dai‐Shi Tian, Chuan Qin

**Affiliations:** ^1^ Department of Neurology Tongji Hospital Tongji Medical College and State Key Laboratory for Diagnosis and Treatment of Severe Zoonotic Infectious Diseases Huazhong University of Science and Technology Wuhan China; ^2^ Hubei Key Laboratory of Neural Injury and Functional Reconstruction Huazhong University of Science and Technology Wuhan China; ^3^ Department of Radiology Tongji Hospital Tongji Medical College Huazhong University of Science and Technology Wuhan China

**Keywords:** bread, diet, Mendelian randomization, multiple sclerosis, oily fish, nested case–control study

## Abstract

Genetic and environmental factors jointly affect the onset of multiple sclerosis (MS), among which diet holds considerable interest as a potentially modifiable factor. A nested case–control study was conducted, including 303 participants with MS and 1212 age‐ and sex‐matched controls from the UK Biobank. Conditional logistic regression models were used to estimate the relationship between diet and MS. Mendelian randomization (MR) analysis was employed to examine the genetic associations between various food types and the risk of MS. Mediation analyses were performed to determine the possible mediating effect of serum measurements using the Karlson–Holm–Breen method. Participants who regularly consumed oily fish and consumed more bread per week had a decreased risk of MS. Increased consumption of oily fish and cereal was genetically associated with a lower risk of MS. The association between oily fish intake and reduced risk of MS remained robust among several subgroups. Besides, vitamin D and neutrophil count mediated the protective effects of oily fish consumption against MS, independently. Increasing the intake of both oily fish and wholemeal/wholegrain bread may reduce the risk of MS onset, while vitamin D and neutrophil count play a partial mediating role during this process.

## Graphical abstract

This study explored the association between dietary habits and the risk of multiple sclerosis (MS) using data from the UK Biobank. A nested case–control design and Mendelian randomization analysis revealed that regular consumption of oily fish and wholemeal or wholegrain bread was linked to a decreased risk of MS. Further mediation analyses indicated that vitamin D levels and neutrophil counts partially mediated the protective effects of oily fish intake. Together, these findings suggest that increasing the intake of oily fish and wholegrain foods may help lower MS risk through both nutritional and immune‐modulating pathways.

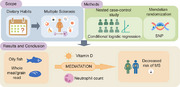

**Abbreviations**
BMI: body mass index
CI: confidence intervals
CNS: central nervous system
EAE: experimental autoimmune encephalitis
IVW: inverse‐variance weighted
KHB: Karlson–Holm–Breen
LMR: lymphocyte‐to‐monocyte ratio
MET: metabolic equivalent task
MR: Mendelian randomization
MS: multiple sclerosis
NLR: neutrophil‐to‐lymphocyte ratio
OR: odds ratios
PLR: platelet‐to‐lymphocyte ratio
PUFA: polyunsaturated fatty acids
RCS: restricted cubic spline
SCFA: short‐chain fatty acids
SII: systemic immune inflammation index
TDI: Townsend deprivation index

## 1 Introduction

Multiple sclerosis (MS) is an immune‐mediated chronic inflammatory demyelinating disease of the central nervous system (CNS), commonly characterized by a relapsing‐remitting clinical course. Clinical symptoms mainly include vision loss, deficits in sensory and motor functions, as well as cognitive impairment. MS primarily affects young individuals, with a higher prevalence among females [1, 2]. Nowadays, there is a better understanding of the underlying genetic and environmental factors that may contribute to the risk of developing MS, such as vitamin D levels, smoking, obesity, and dietary habits [3, 4].

Diet is a potentially modifiable factor in MS onset and progression [5]. Although several studies have explored the importance of dietary habits in MS, robust and consistent evidence is still lacking. Various nutritional patterns, including the Mediterranean, the Paleo, the Swank, the McDougall, the Ketogenic, and the gluten‐free diets have gained popularity [6, 7, 8]. A healthy diet was reported to be associated with reduced disability and less severe symptoms in MS patients [9], and a higher adherence to the Mediterranean diet was associated with lower patient‐reported disability [10]. However, another study showed no significant differences in disease severity among MS patients following different dietary patterns [11]. Nonetheless, recent systematic reviews and meta‐analyses highlighted the complex relationship between diet and MS, mostly focused on general dietary patterns rather than assessing the impact of individual food types [12]. Moreover, large‐scale prospective cohort studies are still lacking.

The UK Biobank is a detailed, long‐term prospective health research study, with participants recruited from 22 assessment centers across the United Kingdom. It gathers information on the consumption frequency of various food types through questionnaires, ensuring highly reproducible dietary data [13]. Leveraging the data from the UK Biobank, our study aimed to assess the association between different food types and the risk of MS. Additionally, we explored the relationship between these food types and the risk of MS at the genetic level using Mendelian randomization (MR) analysis. Moreover, the potential mediating factors were also analyzed to gain further insights into the connection between MS and diet.

## 2 Methods

### 2.1 UK Biobank: Nested Case–Control Study

#### 2.1.1 Study and Participants

We conducted a nested case–control study cohort study based on the UK Biobank, which comprised approximately 500 000 participants aged 37–73 and recruited between 2006 and 2010 from 22 assessment centers across the United Kingdom [14]. UK Biobank has ethical approval from the North‐West Multi‐Center Research Ethics Committee. All participants provided informed consent through formal mail invitations and completed a touch‐screen questionnaire assessing sociodemographic, lifestyle, and physical measurements by themselves at baseline. Well‐trained nurses collected blood samples and the related measurements were obtained during enrollment. More detailed information can be found on the UK Biobank website. The present study was conducted in accordance with the ethical requirements of UK Biobank.

**FIGURE 1:**
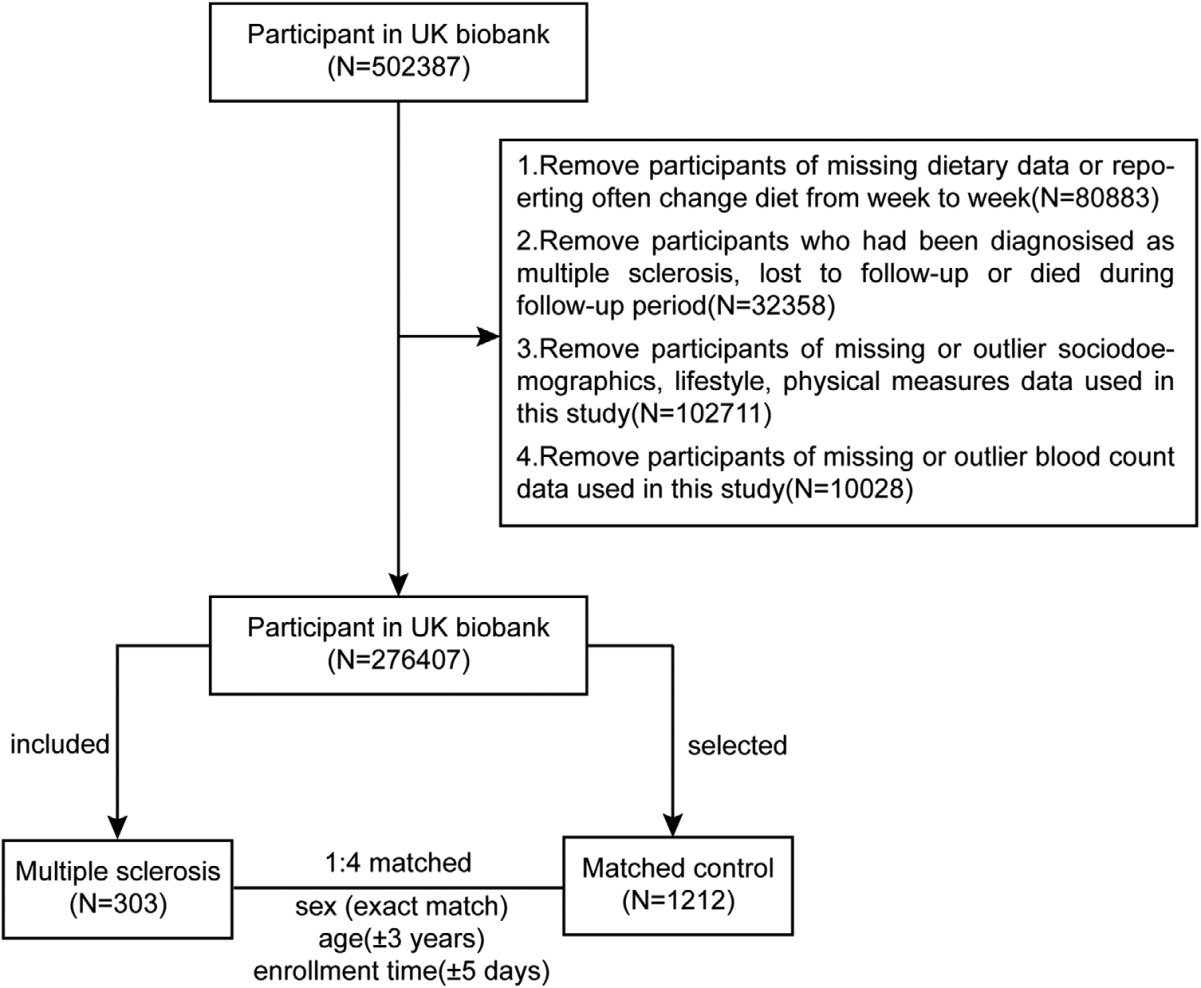
Flowchart of study design. Inclusion and exclusion criteria for participants and the matching procedure for the nested case–control study in UK Biobank.

In the current study, our exclusion criteria were defined as follows (Figure 1): (1) missing dietary data or frequent changes reported in the diet from week to week (*N* = 80 883); (2) prevalent MS diagnosis before baseline and participants who were lost to follow‐up or died during follow‐up period (*N* = 32 358); (3) missing or outlier sociodemographic, lifestyle, physical measurements data used in this study (*N* = 102 711); (4) missing or outlier blood count data (*N* = 10 028). Ultimately, 276 407 participants remained, and 303 of them were diagnosed as incident MS. For each incident MS case, four controls were selected by using the calipmatch package in Stata, which performs nearest‐neighbor matching without replacement. Matching was based on sex (exact match), age (± 3 years), and assessment time (± 5 days). As a result, 303 participants with MS and 1212 matched controls were eventually included in the final analysis (Figure 1).

#### 2.1.2 Assessment of Dietary Intakes

The habitual dietary characteristics concerning major food groups and consumption frequency were obtained through the touchscreen food frequency questionnaire [15]. Relevant questions included the frequency of consumption of cooked vegetable, raw vegetable, fresh fruit, dried fruit, bread, cereal, tea, coffee, water, cheese, oily fish, nonoily fish, poultry, beef, lamb, pork, processed meat, and salt added to food. Individuals were categorized into various groups based on quantitative criteria. Numerical data, including vegetable, fruit, bread, cereal, and drinks were further divided into two categories using the median. While categorical data, such as cheese, fish, and meat consumption, were classified as seldom intake (<1 time/week) and regular intake (≥1 time/week), and individuals were divided into never/rarely and sometimes/usually/always for salt added to food. Participants who answered “Do not know” or “Prefer not to answer” were excluded from this analysis because they could not be properly classified into specific dietary groups.

#### 2.1.3 Assessment of Outcome: Multiple Sclerosis

We defined individuals with MS (data field: 131 042) through mapping information from data in primary care, International Classification of Diseases codes (ICD10:G35) in hospital inpatient data, death register records, and self‐reported medical condition. The endpoint for these analyses was the first diagnosis of MS, the date of death, or the lost or end of the follow‐up (November 12, 2021), whichever came first.

#### 2.1.4 Covariates

Several possible confounding variables were considered as potential covariates, including sociodemographic factors, lifestyle factors, physical measurements, and blood measurements. In specific, covariates were documented, including age at baseline; race (white, non‐white); assessment center; Townsend deprivation index (TDI: calculated from residence data at recruitment and was considered a hallmark of socioeconomic status); body mass index (BMI: calculated as weight divided by squared height kg/m^2^); summed metabolic equivalent task (MET, converted from self‐reported moderate and vigorous physical activity); drinking frequency; and smoking status. The blood measurements, such as vitamin D (potential confounding factors associated with MS), lymphocyte count, monocyte count, platelet count, and neutrophil count, were acquired from the automated quantitative analyzer within 24 h of sample collection at baseline. Comprehensive details are accessible on the online website. The ratios, including neutrophil‐to‐lymphocyte ratio (NLR), platelet‐to‐lymphocyte ratio (PLR), systemic immune inflammation index (SII, neutrophils*platelets/lymphocytes), and lymphocyte‐to‐monocyte ratio (LMR) were further calculated [16].

#### 2.1.5 Statistical Analysis

Regarding the relatively small number of MS participants in the population and UK Biobank, we conducted a nested case–control study in the UK Biobank to investigate the associations between MS onset and the consumption of various types of food, including cooked vegetable, salad raw vegetable, fresh fruit, dried fruit, bread, cereal, tea, coffee, water, cheese, oily fish, nonoily fish, poultry, beef, lamb, pork, processed meat, and salt added to food.

Basic characteristics about sociodemographics, lifestyle, physical measurements, blood measurements, and diet frequency were presented and summarized by case and control status. Extreme outliers (< quartile [Q]1 – 3*interquartile [IQR] or > Q3 + 3*IQR) for numerical index (except age), were excluded from all analyses, and for all numerical index in covariates, centralization, and standardization were performed to eliminate the influence of different scales. Continuous variables are shown as median ± IQR, and categorical variables are shown as numbers (percentages). Mann–Whitney *U*‐test or chi‐squared test were conducted to evaluate the statistical differences at baseline.

Multivariable conditional logistic regression models were performed to explore the associations between diet and MS, and we adjusted the models in several steps with different covariates. Model 1 was adjusted for age, race, and assessment center. Model 2 was additionally adjusted for Townsend deprivation index, MET, and BMI. Model 3 was further adjusted for smoking and drinking status. Results were displayed as odds ratios (ORs) with 95% confidence intervals (CIs).

Several analyses were performed to assess the robustness of our study results. First, we conducted restricted cubic spline (RCS) models to further examine the relationship between oily fish and bread intake and MS. The model was run with four knots at the 5th, 35th, 65th, and 95th percentiles of oily fish and bread intake (reference is the 5th percentile) with adjustment of the range of covariates. Then, we used stratified analyses to examine whether the association between oily fish and bread intake and MS varied by age (< 45, 45–60, ≥ 65 years); sex (male, female); BMI (< 25, ≥ 25 kg/m^2^); smoking (no, yes); drinking (seldom, regular); tertiles for Townsend deprivation index (≤ −3.32, −3.32–0.78, ≥ −0.78); and MET (≤ 1078, 1078–2826, ≥ 2826 min/week). Furthermore, we performed mediation analyses to explore the potential mediators of the relationship between oily fish and bread intake and MS. We estimated the direct, indirect, and total effects of oily fish and bread intake on MS, adjusting covariates. Mediation effects by blood measures were done using the Karlson–Holm–Breen (KHB) method with STATA software. Vitamin D, lymphocyte count, monocyte count, platelet count, neutrophil count, NLR, PLR, SII, and LMR were taken as mediators of the independent variables associated with the risk of MS. The chain mediating roles of vitamin D and neutrophil count on the relationship between oily fish intake were examined by the percentile bootstrap method. Furthermore, we assessed the protective effects of different combinations of bread types and the frequency of oily fish intake on multiple sclerosis. All statistical analyses were performed using R software (version 4.2.1) and STATA (version 17). The *p* values less than 0.05 were assumed to be statistically significant.

#### 2.1.6 Mendelian Randomization Analysis

Two‐sample MR analyses were conducted to explore the genetic associations between various diets and the risk of MS as well as the severity of MS. Effect instrumental variables (SNP) for exposures were used, based on significance threshold (*p* < 5 × 10^−8^), a linkage disequilibrium level of *r* ^2 ^< 0.01, kb = 1000 from corresponding GWAS (https://gwas.mrcieu.ac.uk), *F*‐statistics for each SNP were calculated, and strong instrument variables with *F* > 10 remained. Summary statistics data for MS were extracted from the International Multiple Sclerosis Genetics Consortium [17], and the publicly available GWAS summary statistics for MS severity were obtained and the EDSS score was the indicator for neurological disability [18]. The inverse‐variance weighted (IVW) model was used for the main MR analysis, which generally gave a consistent estimate of the causality with the most power. Four additional methods were conducted to prove the robustness of the results, including MR Egger, weighted median, simple mode, and weighted model. MR‐PRESSO was further executed to recognize and eliminate outliers to provide more accurate results. Results were generated after removing outliers. Sensitivity analysis included a heterogeneity test, and a pleiotropy test was also conducted. The intercept of MR‐Egger regression provided an assessment of horizontal pleiotropy, and Cochran's Q statistic inspected the heterogeneity across all instrumental SNPs. Statistical significance was set at a *p* < 0.05.

## 3 Results

### 3.1 Baseline Characteristics of Participants

At baseline, 502 387 participants were initially assessed in the UK Biobank. Following the exclusion criteria outlined in Figure 1, a total of 303 newly diagnosed MS cases and 1212 matched controls were included in the final analysis.

The baseline characteristics of the participants are presented in Table 1. Overall, there were no significant difference in terms of age and sex between participants with and without MS. However, individuals with MS were found to be less physically active, as shown by lower summed MET (*p* = 0.046), and had a higher ratio of smoking history (*p* = 0.002). Blood measurements indicated that MS cases had significantly lower vitamin D levels (*p* < 0.001) and a higher neutrophil count (*p* < 0.001). Additionally, as detailed in Table 2, the diet habits of the participants revealed that MS patients tended to consume less than 10 slices of bread per week (*p* = 0.017) and were less likely to regularly intake oily fish (*p* = 0.003) compared to participants without MS. Given the potential influence of demographic factors on diet habits, we examined these associations and observed that diet habits differed by age, sex, race, region, and socioeconomic status, with detailed associations presented in Table S1.

**TABLE 1:** Baseline characteristics of the study participants in the UK Biobank (*N* = 1515).

	Matched controls (*n* = 1212)	Multiple Sclerosis cases (*n* = 303)	*p* value
Age, y	52 (46, 60)	52 (46, 60)	0.720
Sex, %			1
Male	452 (37.29)	113 (37.29)	
Female	672 (55.45)	190 (62.71)	
Race, %			0.061
White	68 (5.61)	9 (2.97)	
Non‐White	1144 (94.39)	294 (97.03)	
Townsend deprivation index	−2.41 (−3.83, 0.26)	−2.24 (−3.61, 0.31)	0.416
BMI, kg/m^2^			0.258
< 25	483 (39.85)	110 (36.30)	
≥ 25	729 (60.15)	193 (63.70)	
Summed MET, min/week	1836.75 (855.00, 3471.75)	1573.00 (555.50, 3646.00)	0.046
Ever smoking, %			0.002
No	540 (44.55)	105 (34.65)	
Yes	672 (55.45)	198 (65.35)	
Alcohol intake frequency, %			0.052
Never/occasionally	206 (17.00)	66 (21.78)	
Regular	1006 (83.00)	237 (78.22)	
Vitamin D, nmol/L	48.45 (32.90, 63.20)	39.00 (27.45, 56.45)	< 0.001
Lymphocyte count, 10^9^/L	1.85 (1.50, 2.20)	1.90 (1.50, 2.34)	0.175
Monocyte count, 10^9^/L	0.43 (0.35, 0.54)	0.45 (0.36, 0.58)	0.221
Platelet count, 10^9^/L	251.75 (251.98, 292.00)	260.80 (218.75, 295.45)	0.146
Neutrophil count, 10^9^/L	4.00 (3.20, 4.88)	4.20 (3.50, 5.31)	< 0.001
NLR	2.17 (1.71, 2.77)	2.29 (1.73, 2.89)	0.134
PLR	139.47 (109.65, 171.44)	136.05 (108.10, 173.20)	0.708
SII	559.88 (407.13, 713.85)	582.90 (420.11, 781.33)	0.077
LMR	4.19 (3.29, 5.33)	4.20 (3.26, 5.57)	0.896

*Note*: Continuous variables are shown as medians and inter‐quartile ranges, and categorical variables are shown as numbers (percentages).

Abbreviations: BMI, body mass index; LMR, lymphocyte‐to‐monocyte ratio; MET, metabolic equivalent task; NLR, neutrophil‐to‐lymphocyte ratio; PLR, platelet‐to‐lymphocyte ratio; SII, systemic immune‐inflammation index.

**TABLE 2:** Diet characteristics of the study participants in the baseline (*N* = 1535).

	Matched controls (*n* = 1212)	Multiple sclerosis cases (*n* = 303)	*p* value
Cooked vegetable intake, tablespoons/day	2 (2, 3)	2 (2, 3)	0.323
Cooked vegetable intake group, %			0.279
≤ 2	638 (52.64)	170 (56.11)	
> 2	574 (47.36)	133 (43.89)	
Salad raw vegetable intake, tablespoons/day	2 (1, 3)	2 (1, 3)	0.284
Salad raw vegetable intake group, %			0.978
≤ 2	825 (68.07)	206 (67.99)	
> 2	387 (31.93)	97 (32.01)	
Fresh fruit intake, pieces/day	2 (1, 3)	2 (1, 3)	0.561
Fresh fruit intake group, %			1
≤ 2	768 (63.37)	192 (63.37)	
> 2	444 (36.63)	111 (36.63)	
Dried fruit intake, pieces/day	0.5 (0, 1)	0 (0, 1)	0.157
Dried fruit intake group, %			0.106
= 0	597 (49.26)	165 (54.46)	
> 0	615 (50.74)	138 (45.54)	
Bread intake, slices/week	10 (6, 16)	10 (6, 14)	0.039
Bread intake group, %			0.017
≤ 10	635 (52.39)	182 (60.07)	
> 10	577 (47.61)	121 (39.93)	
Cereal intake, bowls/week	5 (2, 7)	5 (2, 7)	0.475
Cereal intake group, %			0.536
≤ 5	644 (53.14)	167 (55.12)	
> 5	568 (46.86)	136 (44.88)	
Tea intake, cups/day	3 (1, 5)	3 (1, 5)	0.611
Tea intake group			0.533
≤ 3	696 (57.43)	168 (55.45)	
> 3	516 (42.57)	135 (44.55)	
Coffee intake, cups/day	1 (0.5, 3)	1 (0, 3)	0.304
Coffee intake group			0.484
≤ 2	853 (70.38)	207 (68.32)	
> 2	359 (29.62)	96 (31.68)	
Water intake, glasses/day	2 (1, 4)	2 (1, 4)	0.705
Water intake group, %			0.328
≤ 2	630 (51.98)	167 (55.12)	
> 2	582 (48.02)	136 (44.88)	
Cheese intake, %			0.315
Seldom	243 (20.05)	53 (17.49)	
Regular	969 (79.95)	250 (82.51)	
Oily fish intake, %			0.003
Seldom	514 (42.41)	157 (51.82)	
Regular	698 (57.59)	146 (48.18)	
Non‐oily fish intake, %			0.870
Seldom	406 (33.50)	100 (33.00)	
Regular	806 (66.50)	203 (66.99)	
Poultry intake, %			0.778
Seldom	192 (15.84)	46 (15.18)	
Regular	1020 (84.16)	257 (84.82)	
Beef intake, %			0.437
Seldom	674 (55.61)	176 (58.09)	
Regular	538 (44.39)	127 (41.91)	
Lamb intake, %			0.235
Seldom	939 (77.48)	225 (74.26)	
Regular	273 (22.52)	78 (25.74)	
Pork intake, %			0.210
Seldom	922 (76.07)	220 (72.61)	
Regular	290 (23.93)	83 (27.39)	
Processed meat intake, %			0.603
Seldom	512 (42.24)	123 (40.59)	
Regular	700 (57.76)	180 (59.41)	
Salt added to food, %			0.854
Never/rarely	725 (59.82)	183 (60.40)	
Sometimes/ usually/ always	487 (40.18)	120 (39.60)	

*Note*: Values are presented as medians and inter‐quartile ranges for continuous variables and as number (percentage) for categorical variables.

### 3.2 Associations Between Diet and Risk of Multiple Sclerosis

**FIGURE 2:**
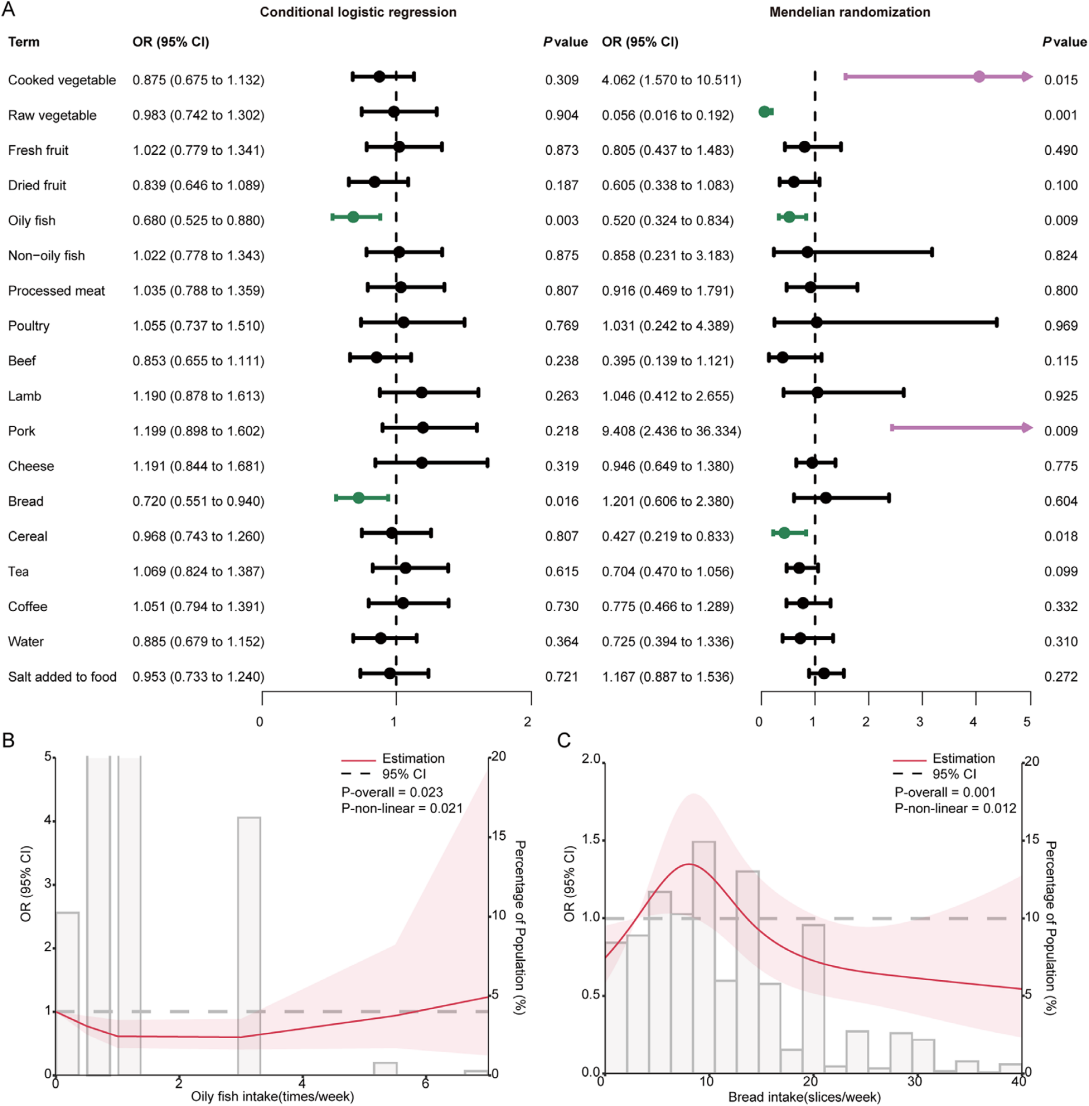
Associations between diet and the risk of multiple sclerosis. (A) Exploration of the relationship between 18 types of diet and multiple sclerosis in conditional logistic regression analysis with fully adjusted and Mendelian randomization analysis. Dots represent odds ratios and lines horizontal represent corresponding 95% confidence intervals. (B) The associations between and frequency of oily fish intake and multiple sclerosis using a restricted cubic spline model with fully adjusted. (C) The associations between bread intake and multiple sclerosis using restricted cubic spline model with fully adjusted. Full adjustment: adjusted for age, race, assessment center, Townsend deprivation index, MET, BMI, drinking frequency and smoking status. CI indicates confidence intervals; OR, odds ratio.

To further investigate the association between diet habits and risk of MS, we then conducted conditional logistic regression analysis, with regular indexes like age, race, assessment center, Townsend deprivation index, drinking and smoking status, MET, and BMI which showed differences between the MS patients and controls as confounding variables. In the conditional logistic regression model, after adjusting, we identified a statistically significant association between oily fish intake and risk of MS (OR, 0.680; 95% CI, 0.525–0.880; *p* = 0.003), and a notable association between bread consumption and the onset of MS (OR, 0.720; 95% CI, 0.551–0.940; *p* = 0.016) (Figure 2A). Detailed results of the non‐significant association between diet and the risk of MS through conditional logistic regression were shown in Table S2.

To explore the genetically causal effects of different dietary factors on the risk of MS, two‐sample MR analyses were performed. The results suggested that increased consumption of raw vegetables (OR, 0.056; 95% CI, 0.016–0.192; *p* = 0.001); oily fish (OR, 0.520; 95% CI, 0.324–0.834; *p* = 0.009); and cereal (OR, 0.427; 95% CI, 0.219–0.833; *p* = 0.018) were associated with a lower risk of MS, while a higher intake of cooked vegetables (OR, 4.062; 95% CI, 1.570–10.511; *p* = 0.015) and pork (OR, 9.408; 95% CI, 2.436–36.334; *p* = 0.009) increases MS risk (Figure 2A). The effect estimates of oily fish and cereal were consistent using alternative MR methods, including MR Egger, weighted median, simple mode, and weighted mode (Table S3). Additionally, we evaluated the association of diet with the severity of MS through MR. There was no causal relationship between diet and the severity of MS (Table S4, Figure S1).

To further examine the relationship between oily fish and bread intake and MS, we conducted restricted cubic spline models separately. In the fully adjusted model, the associations of oily fish (*p* = 0.021) and bread intake (*p* = 0.012) with MS were found to be nonlinear (Figure 2B,C).

### 3.3 Stratified Analysis of the Relationship Between Diet and Multiple Sclerosis

**FIGURE 3:**
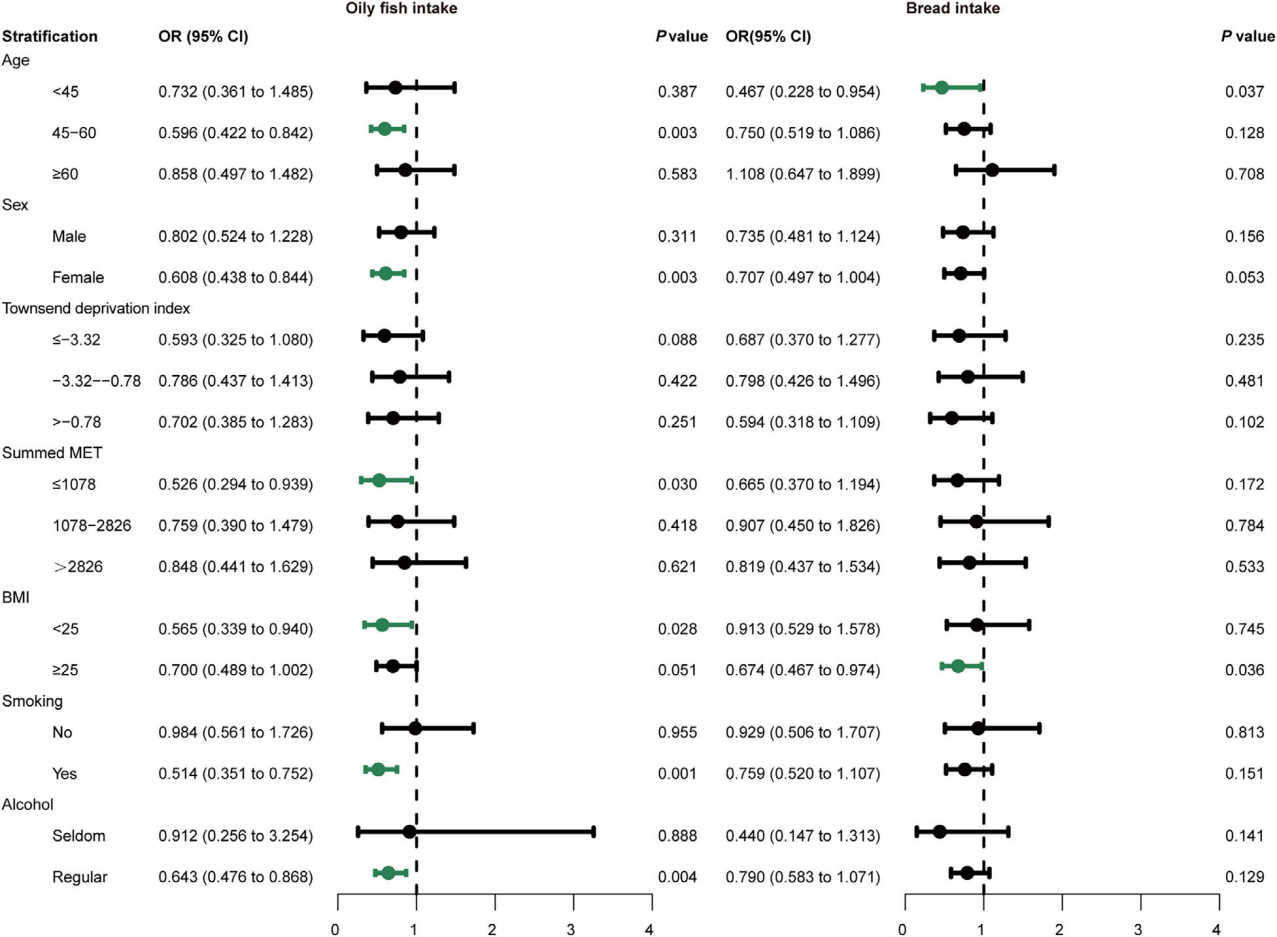
Stratified analysis of the relationship between oily fish and bread intake and multiple sclerosis. Forest plots of stratified analysis for the association between oily fish and bread intake and multiple sclerosis. BMI indicates body mass index; MET, metabolic equivalent task.

We performed a stratified analysis to further investigate the relationship between diet and MS across subgroups. As shown in Figure 3, the association between oily fish and bread intake and a reduced risk of multiple sclerosis remains robust in certain groups.

For oily fish intake, we observed that individuals aged 45–60 and females exhibited a reduced risk of MS (OR, 0.596; 95% CI, 0.422–0.842; *p* = 0.003; OR, 0.608; 95% CI, 0.438–0.844; *p* = 0.003, respectively). A robust association was also observed among participants with less summed MET (≤ 1078 min/week) (OR, 0.526; 95% CI, 0.294–0.939; *p* = 0.030) and lower body mass index (< 25 kg/m^2^) (OR, 0.565; 95% CI, 0.339–0.940; *p* = 0.028). In addition, consistent results were observed for cigarette and alcohol consumption, indicating that regular intake of oily fish may decrease the risk of MS particularly in individuals who had ever smoked (OR, 0.514; 95% CI, 0.351–0.752; *p* = 0.001) and were regular alcohol consumers (OR, 0.643; 95% CI, 0.476–0.868; *p* = 0.004). Meanwhile, the stratified analysis of bread intake and MS revealed that younger individuals (< 45 years) (OR, 0.467; 95% CI, 0.228–0.954; *p* = 0.037) and those with a higher body mass index (≥ 25 kg/m^2^) (OR, 0.674; 95% CI, 0.467–0.974; *p* = 0.036) tended to exhibit a reduced risk of MS (Figure 3).

### 3.4 Mediation Analysis of the Relationship Between Oily Fish and Bread Intake and MS

**FIGURE 4:**
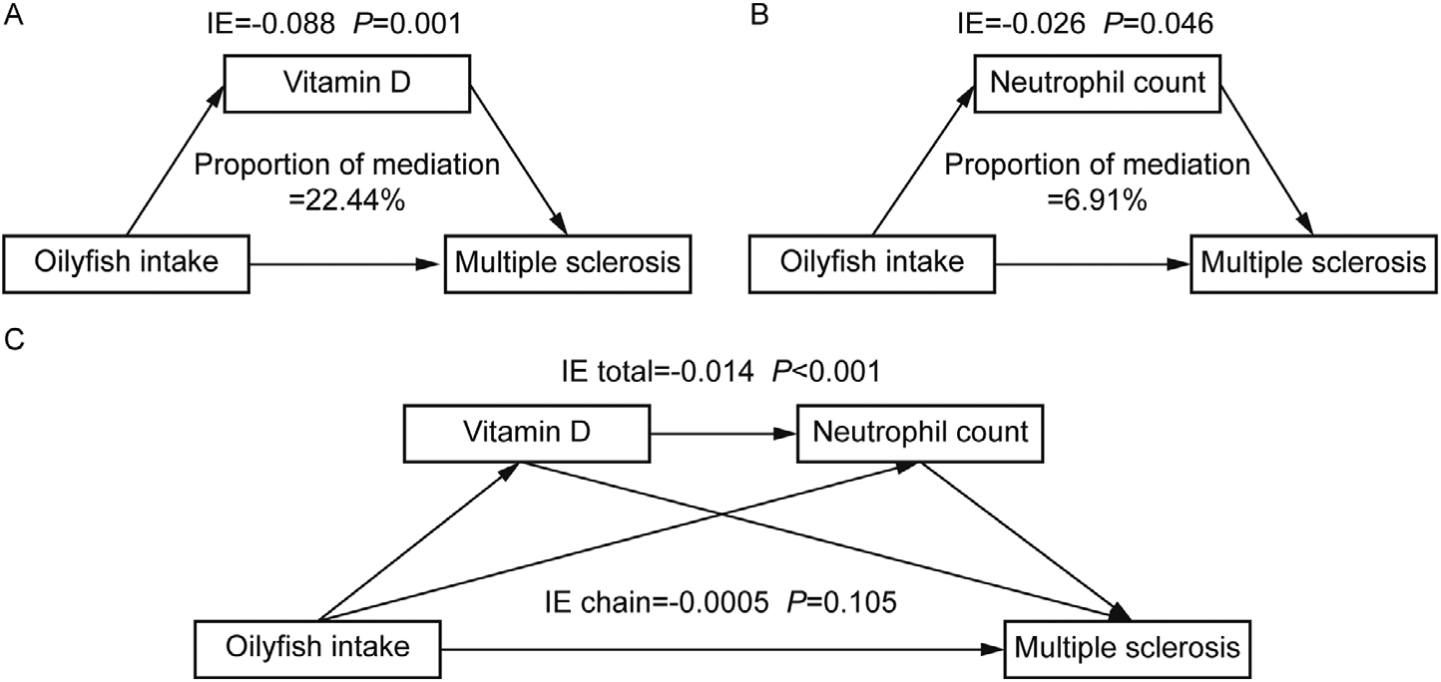
Mediation analysis of the effect of oily fish intake on multiple sclerosis. The relationship between oily fish and MS was partially mediated by vitamin D and neutrophil count. Results were adjusted for age, race, assessment center, Townsend deprivation index, MET, BMI, drinking frequency, and smoking status. IE indicates indirect effect.

We performed mediation analyses to explore the potential mediators in the relationship between oily fish and bread intake and MS (Table S5). Based on the analysis of baseline characteristics, MS participants displayed relatively lower vitamin D levels and higher neutrophil counts. This prompted us to explore whether the vitamin D level and neutrophil count play mediating roles in the relationship. After controlling for covariates, the mediation analyses revealed that both vitamin D and neutrophil count mediated the association between oily fish intake and the risk of MS. In detail, the indirect effect through vitamin D was −0.088 (*p* = 0.001) with a mediation proportion of 22.44% (Figure 4A). Similarly, the neutrophil count showed 6.91% of the mediating effect, with an indirect effect of −0.026 (*p* = 0.046) (Figure 4B). Besides, we also explored the mediating role of other serum measurements in the relationship between oily fish as well as bread intake and MS risk (Figure S2, Table S5); however, the results showed no significant mediating effect. Furthermore, we examined the mediating effect of vitamin D and neutrophil count between oily fish intake and bread intake and MS through MR. The analysis revealed the indirect effect through vitamin D was −0.048 (*p* = 0.005) (Figure S3A), while no significant effect was observed through neutrophil count (Figure S3B,C).

Then, we conducted the chain‐mediating analysis to explore whether vitamin D and neutrophil exert the separate mediating effects in the relationship between oily fish and MS (Figure 4C). The findings indicated that the total indirect effect was −0.014 (*p* < 0.001), whereas the chain indirect effect was −0.0005 (*p* = 0.105), showing independent mediating effects of vitamin D and neutrophil count.

### 3.5 Association Between Bread Type and MS

Various types, such as white bread, brown bread, wholemeal or wholegrain, and other types, were considered in the UK Biobank. Considering the interaction between bread intake level and bread type, a multivariable regression model was conducted to investigate which factor played the predominant role. In the model adjusting for age, race, assessment center (Model 1), both bread intake level (OR, 0.662; 95% CI, 0.448–0.978; *p* = 0.038) and bread type (OR, 0.648; 95% CI, 0.455–0.922; *p* = 0.016) show protective effect against MS onset. Similar results (OR, 0.674; 95% CI, 0.456–0.997; *p* = 0.048) (OR, 0.662; 95% CI, 0.464–0.943; *p* = 0.022) were found in Model 2, which additionally adjusted for Townsend deprivation index, MET, and BMI. It is worth noting that, in the fully adjusted model, only wholemeal/wholegrain bread intake still exhibited a beneficial influence on lowering the risk of MS (OR, 0.690; 95% CI, 0.482–0.988; *p* = 0.043) (Table 3). The findings suggested that the beneficial impact of consuming wholemeal/wholegrain bread remained robust in preventing MS. Additionally, we examined the potential mediators in the relationship between bread type and MS through mediation analysis. But no significant results were discovered in these analyses (Figure S4, Table S5). We also evaluated the genetical mediating role of neutrophil count in the relationship between bread type and MS, and the result showed no significant mediation effect (Figure S3D). Therefore, comparing to increasing the quantity of bread intake, switching diet type to intake wholemeal/wholegrain bread was more beneficial for the prevention of MS.

**TABLE 3:** Association between bread type and multiple sclerosis.

Predictor	Model 1		Model 2		Model 3	
	OR (95% CI)	*p* value	OR (95% CI)	*p* value	OR (95% CI)	*p* value
Bread type: wholemeal or wholegrain	0.648 (0.455, 0.922)	0.016	0.662 (0.464, 0.943)	0.022	0.690 (0.482, 0.988)	0.043
Bread intake	0.662 (0.448, 0.978)	0.038	0.674 (0.456, 0.997)	0.048	0.687 (0.463, 1.019)	0.062
Bread type* bread intake	1.004 (0.590, 1.706)	0.989	1.000 (0.587, 1.702)	0.999	0.963 (0.565, 1.644)	0.891

*Note*: Model 1: Adjusted for age, race, and assessment center. Model 2: Based on Model 1 and additionally adjusted for Townsend deprivation index, MET, and body mass index. Model 3: Based on Model 2 and further adjusted for drinking and smoking status.

Abbreviations: CI, confidence intervals; OR, odd ratio.

### 3.6 The Effect of the Combination of Bread Type and Oily Fish Intake on Multiple Sclerosis

**FIGURE 5:**
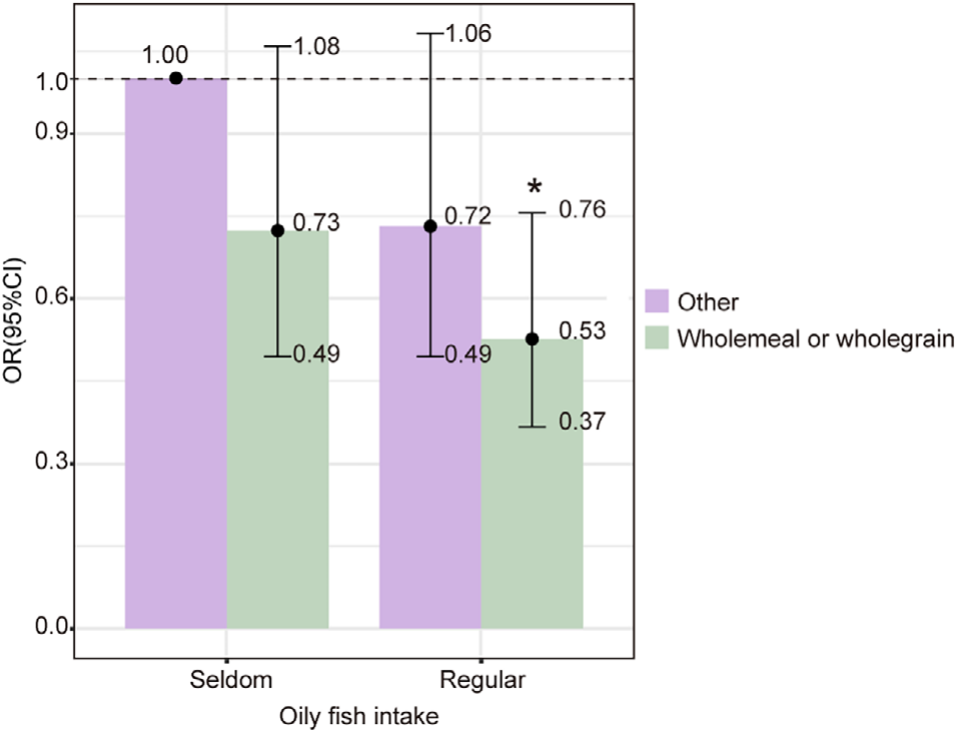
The effect of the combination of bread type and oily fish intake on multiple sclerosis. Multivariable models adjusted for age, race, assessment center, Townsend deprivation index, MET, BMI, drinking frequency, and smoking status. CI indicates confidence intervals; OR, odd ratio. *p < 0.05. Statistical significance was evaluated using conditional logistic regression with Wald test.

Given the identified protective effect of oily fish and wholemeal/wholegrain bread intake, we further explored the combined dietary pattern of the two for its protective effects on MS. In multivariable models adjusted for age, race, assessment center, Townsend deprivation index, MET, BMI, drinking frequency, and smoking status, participants who consumed both oily fish and wholemeal/wholegrain bread exhibited a 47% reduced likelihood of having MS (OR = 0.527; 95% CI, 0.367–0.756; *p* < 0.001) and experienced significant benefits compared to those who consumed oily fish and wholemeal/wholegrain bread separately (Figure 5). Our results suggested that combined intake of oily fish and wholemeal/wholegrain bread exhibited a more beneficial dietary pattern for MS.

## 4 Discussion

In this current nested case–control study based on the large prospective cohort, including 303 participants with newly diagnosed MS and 1212 matched controls, we aimed to explore the relationship between diet and the risk of MS. Our findings revealed that regular intake of oily fish and consumption of whole meal/whole grain bread are associated with a decreased risk of MS. In addition, we verified through MR analysis that regular intake of oily fish and cereal exert protective effects against the onset of MS genetically. Lastly, we also showed that vitamin D and neutrophil count play a crucial role in mediating the relationship between oily fish intake and the onset of MS. Taken together, these findings suggest that a diet comprising a combination of oily fish and wholemeal/whole grain bread may notably reduce the risk of developing MS.

MS is an autoimmune disease of the CNS, characterized by inflammatory infiltration and demyelination [19]. Neutrophils and monocytes, acting as immune defense cells, are recruited to sites of neuroinflammation during the pathological progression of MS [20]. Previous studies have demonstrated that specific dietary habits, known as pro‐inflammatory diet, could trigger the production of inflammatory factors, including TNFα, IL‐2, MMP9, and prostaglandins, thereby leading to inflammatory damage and oxidative stress [7, 21]. Several studies have reported a relationship between dietary inflammatory index and antioxidant capacity and autoimmune demyelinating diseases, especially MS [22, 23]. However, the association between diet and inflammation in MS remains unclear.

Oily fish refers to fish with high levels of fat in their soft tissues and intestines, such as sardines, salmon, mackerel, and herring, among others. The types of fish are abundant in fatty acids, particularly omega‐3 polyunsaturated fatty acids (PUFAs) [24, 25]. The consumption of omega‐3 PUFAs has been found to reduce the severity of demyelination and neurodegeneration in MS [8]. However, current research findings on the impact of omega‐3 PUFAs in MS remain inconsistent. One previous study involving participants from the Nurses’ Health Study cohorts, where 195 new cases of MS were identified among close to 200 000 individuals, claimed no significant correlation between the intake of omega‐3 fatty acids and the occurrence of MS [26]. While another prospective study, identifying 479 new cases of MS, reported a lower incidence of MS among individuals consuming a diet rich in PUFAs [27]. In the experimental autoimmune encephalitis (EAE) model, oral administration of downstream metabolites of omega‐3 PUFAs reduced MS progression by suppressing autoreactive T cells and skewing microglia toward an anti‐inflammatory phenotype [28]. In another skin inflammation model in mice, PUFAs can alleviate the inflammatory response by inhibiting neutrophil infiltration [29]. Thus, we speculated that oily fish rich in PUFAs may exert a protective effect on MS by suppressing inflammatory responses mediated by neutrophils.

In addition to being rich in omega‐3 PUFAs, oily fish is also a good source of fat‐soluble vitamins, such as vitamin D [30]. Patients with MS are often accompanied by vitamin D deficiency, and the crucial role of vitamin D in the pathogenesis of MS has been confirmed [31, 32, 33, 34]. Sufficient vitamin D levels may be an important modifiable risk factor for MS [35]. However, a systematic review of the influence of diet on MS revealed inconsistencies regarding the association between vitamin D and MS, particularly in relation to disability level and MRI lesions [36, 37]. Nevertheless, vitamin D plays a key role in immune regulation and reducing oxidative stress [7]. Vitamin D supplementation has been shown to have anti‐inflammatory and immunomodulatory effects on the pathogenesis of MS by inhibiting the production of CD4^+^ T cells, thereby reducing the risk of MS and slowing disease progression [38].

Considering both factors, the protective effect of consuming oily fish could be attributed to the influence of omega‐3 PUFAs and vitamin D on the inflammatory response. However, our additional mediation effect analysis illustrated the vitamin D and neutrophil count only partially mediated the protective effects. Hence, increasing consumption of oily fish could help preventing the onset of MS, while solely supplementation of omega‐3 PUFAs or vitamin D cannot substitute for the intake of oily fish.

Cereal and grain are essential components of a heathy diet. Several studies have proved that whole grain intake is associated with a lower risk of cardiovascular disease and type 2 diabetes [39, 40]. However, work on the relationship between grain bread intake and MS is currently limited. A retrospective study reported that individuals who consumed less wholegrain bread and exhibited poorer diet quality during childhood had an increased risk of developing MS; however, the accuracy of the results may be influenced by a single‐center design and recall bias [6]. Increased whole grain intake may confer protective effects by regulating glucose metabolism levels, suppressing inflammation and oxidative stress [41, 42], and modulating immune responses through the production of short‐chain fatty acids (SCFAs) via dietary fiber fermentation [8]. In the EAE model, exogenous administration of SCFA can improve the course of MS by promoting the expression of anti‐inflammatory factors and the differentiation of T cells [43]. Consequently, the fermentation process that occurs after consuming cereal, especially whole wheat bread, leading to the production of SCFAs, may exert anti‐inflammatory protective effects in MS. Our results demonstrated the importance of consuming wholemeal/wholegrain bread in the nested case–control study, and the MR analysis indicated a protective effect of increased intake of cereal on MS. Although the food types are not completely consistent, the results jointly suggested the protective role of grain ingredients in MS.

Our study has several innovative strengths. Firstly, the association between various diet types and the risk of MS was systematically and comprehensively explored based on a large prospective population‐based cohort. MR analysis was also conducted to confirm the role of diet on the onset of MS at a genetic level. Through mediation effect analysis, we elucidated the significant role of vitamin D and neutrophil count in mediating the impact of oily fish intake on the onset of MS. This provides a scientific basis for advocating regular consumption of oily fish as a preventive measure against MS. Finally, we proposed that increasing the intake of oily fish and wholegrain bread simultaneously may effectively reduce the risk of MS, offering a new dietary pattern for MS prevention. However, there are still several limitations to our study that should be noted. The absence of detailed information about the frequency and duration of oily fish intake hindered us from further assessing the dose‐response relationship. Additionally, since the majority of the UK Biobank data are composed of white ethnicity (95.43%), the relationship between diet and MS observed in our study may not fully represent the global population. Further studies are warranted to elucidate the underlying mechanisms.

## 5 Conclusion

In conclusion, our study provided evidence that regular consumption of oily fish and wholemeal/wholegrain bread can effectively lower the risk of developing MS, verifying the association between various diets patterns and the onset of MS at both the epidemiological and genetic levels. Furthermore, we identified the pivotal roles of vitamin D and neutrophil count in mediating the protective effects of oily fish intake against MS. Additionally, our findings suggest that increasing the intake of both oily fish and wholemeal/wholegrain bread may have a synergistic effect in reducing the risk of MS, while further research is required to explore the underling mechanisms.

## Funding

This study was funded by National Natural Science Foundation of China (Grants: 82371404, 82301510, 82271341, and 82071380), Knowledge Innovation Program of Wuhan Shuguang Project (2022020801020454), Tongii Hospital (HUST) Foundation for Excellent Young Scientist (24‐2KYC1305712), and Hubei Provincial Natural Science Foundation Joint Fund for Innovation and Development Project (No. 2023AFD046).

## Conflicts of Interest

The authors declare no conflicts of interest.

## Supporting information




**Supporting file 1**: mnfr70313‐sup‐0001‐tablesS1‐S5.xlsx

**Supporting file 2**: mnfr70313‐sup‐0002‐figureS1.tif

**Supporting file 3**: mnfr70313‐sup‐0003‐figureS2.tif

**Supporting file 4**: mnfr70313‐sup‐0004‐figureS3.tif

**Supporting file 5**: mnfr70313‐sup‐0005‐figureS4.tif

## Data Availability

The present study was performed based on the UK Biobank application number 94533. These data were derived from the following resources available in the public domain.
