# Supplementary figures and images for: Diet and the Risk of Multiple Sclerosis: Evidence With UK Biobank Nested Case–Control Study and Mendelian Randomization Analysis
Source: Mol Nutr Food Res. 2025 Nov 21;69(24):e70313. doi: 10.1002/mnfr.70313 (PMC12700044; doi:10.1002/mnfr.70313)

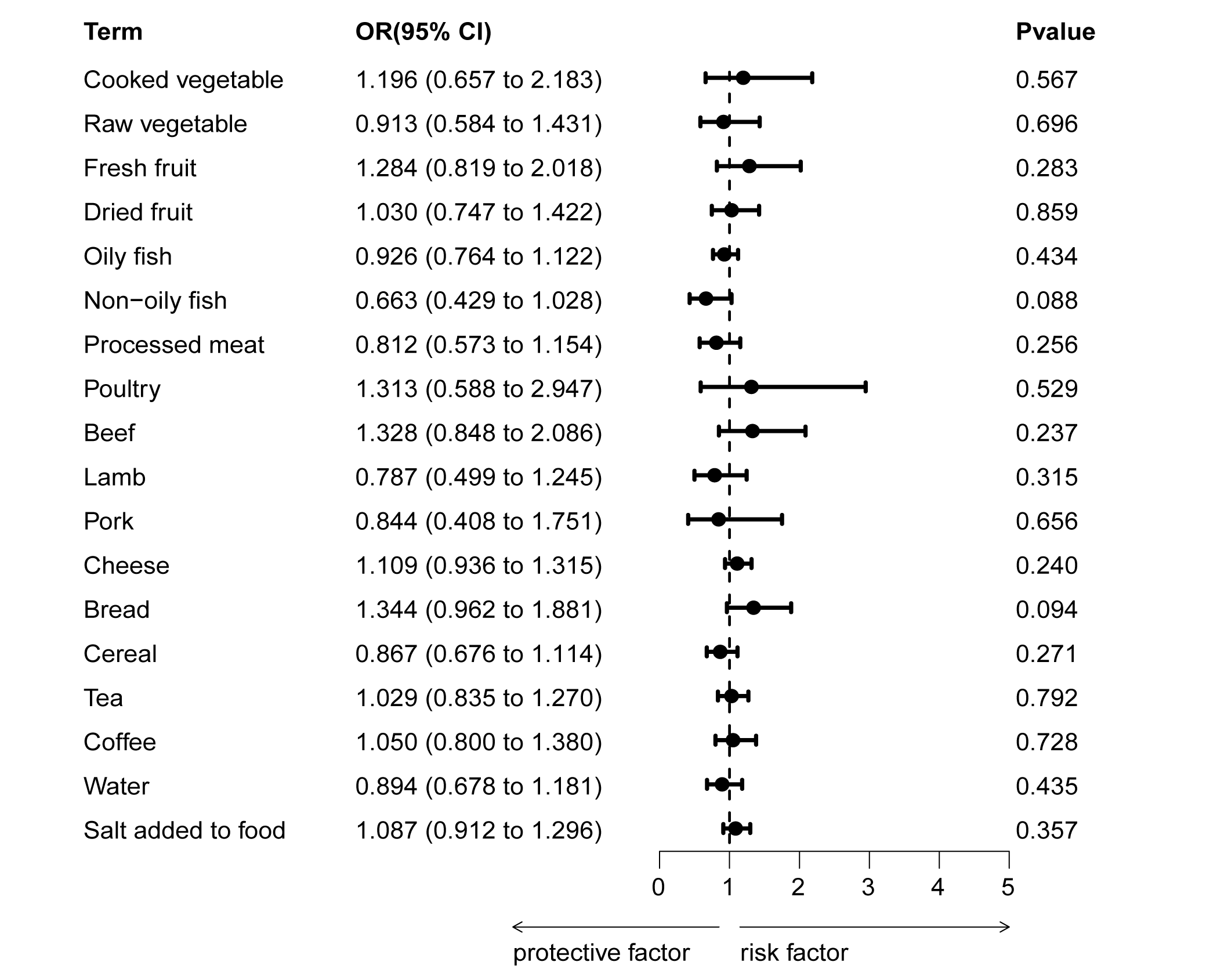

Supplement: Supplementary file 2 — Supporting file 2: mnfr70313‐sup‐0002‐figureS1.tif [file MNFR-69-e70313-s005.tif]

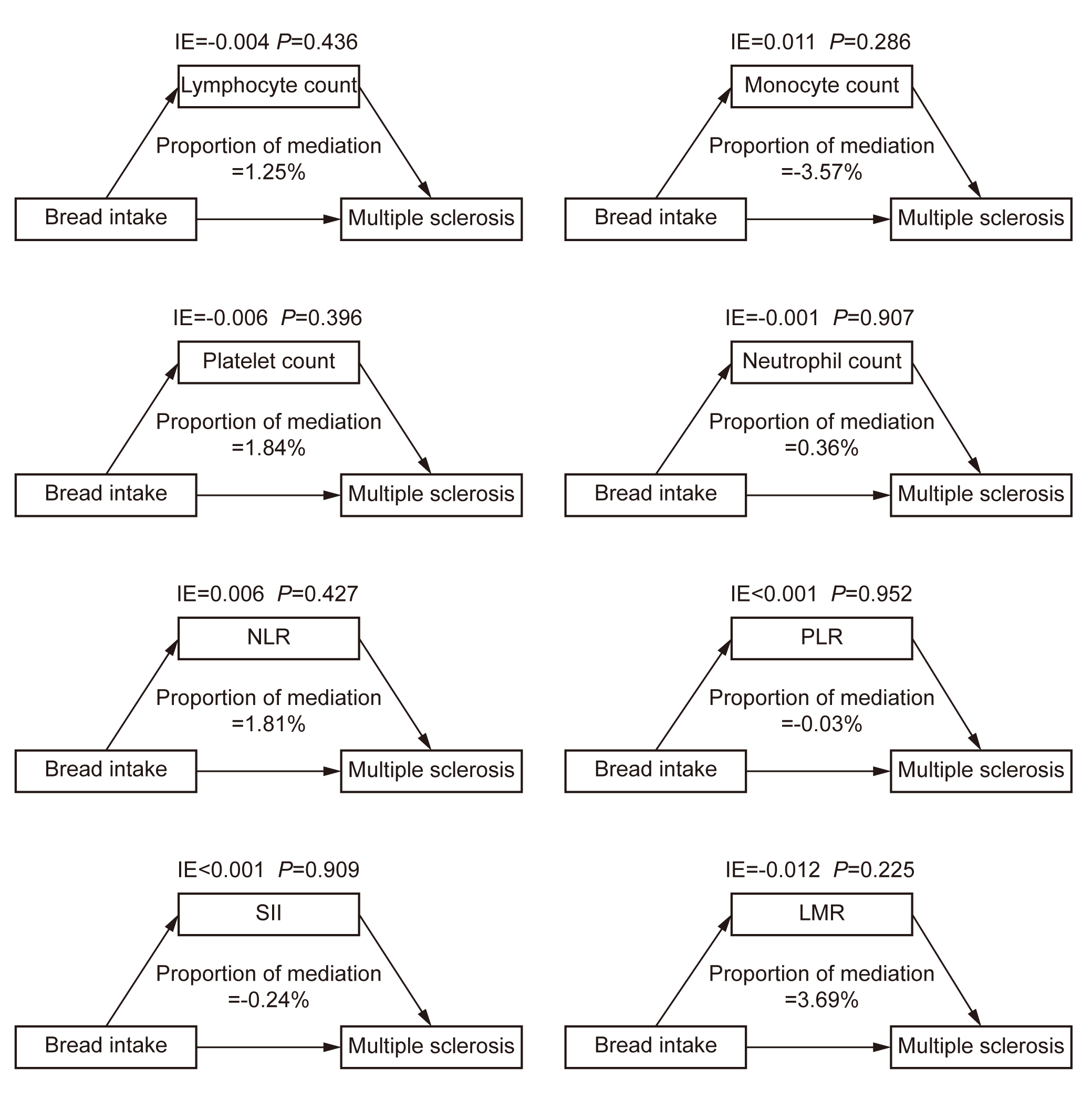

Supplement: Supplementary file 3 — Supporting file 3: mnfr70313‐sup‐0003‐figureS2.tif [file MNFR-69-e70313-s002.tif]

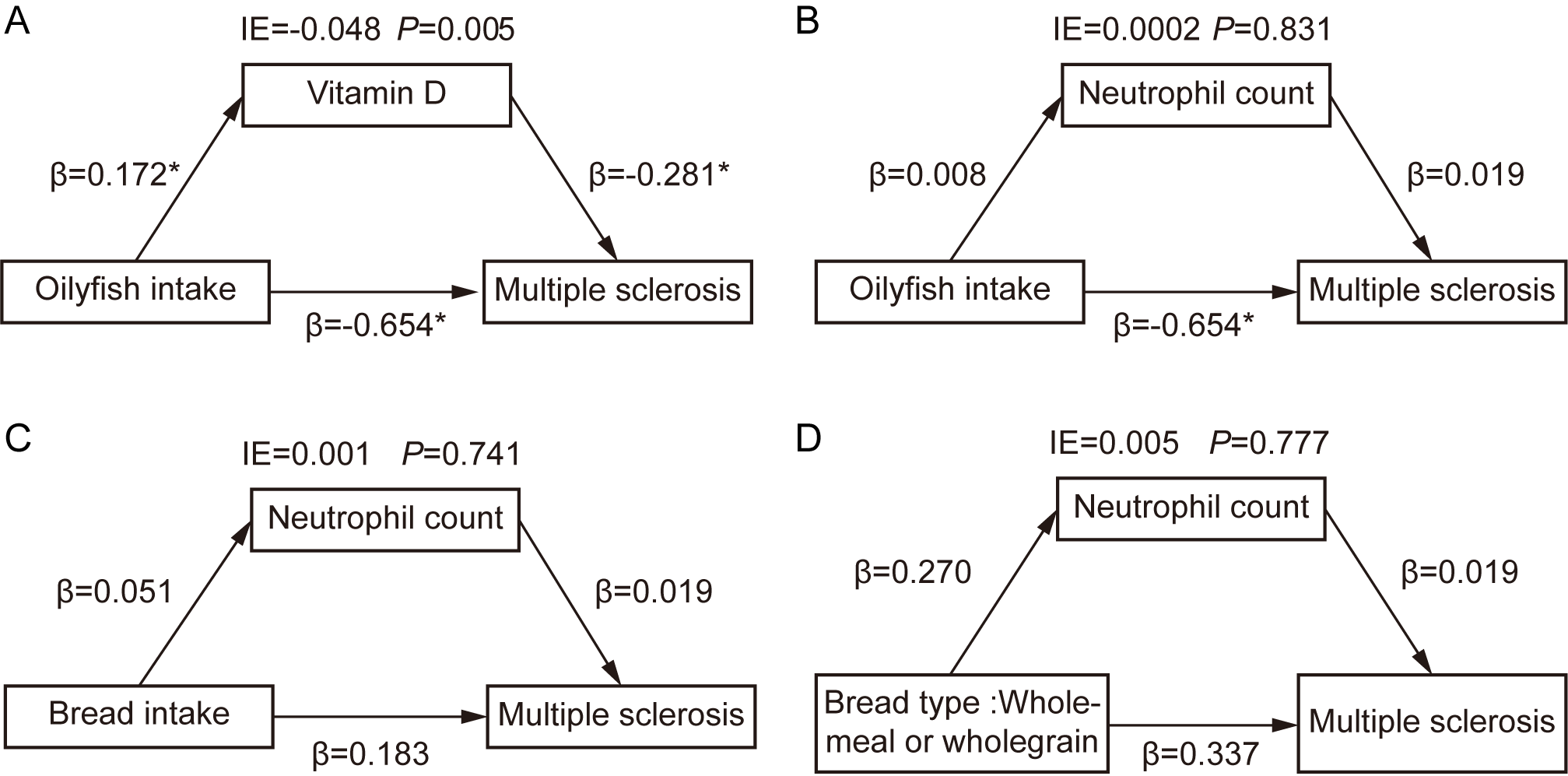

Supplement: Supplementary file 4 — Supporting file 4: mnfr70313‐sup‐0004‐figureS3.tif [file MNFR-69-e70313-s003.tif]

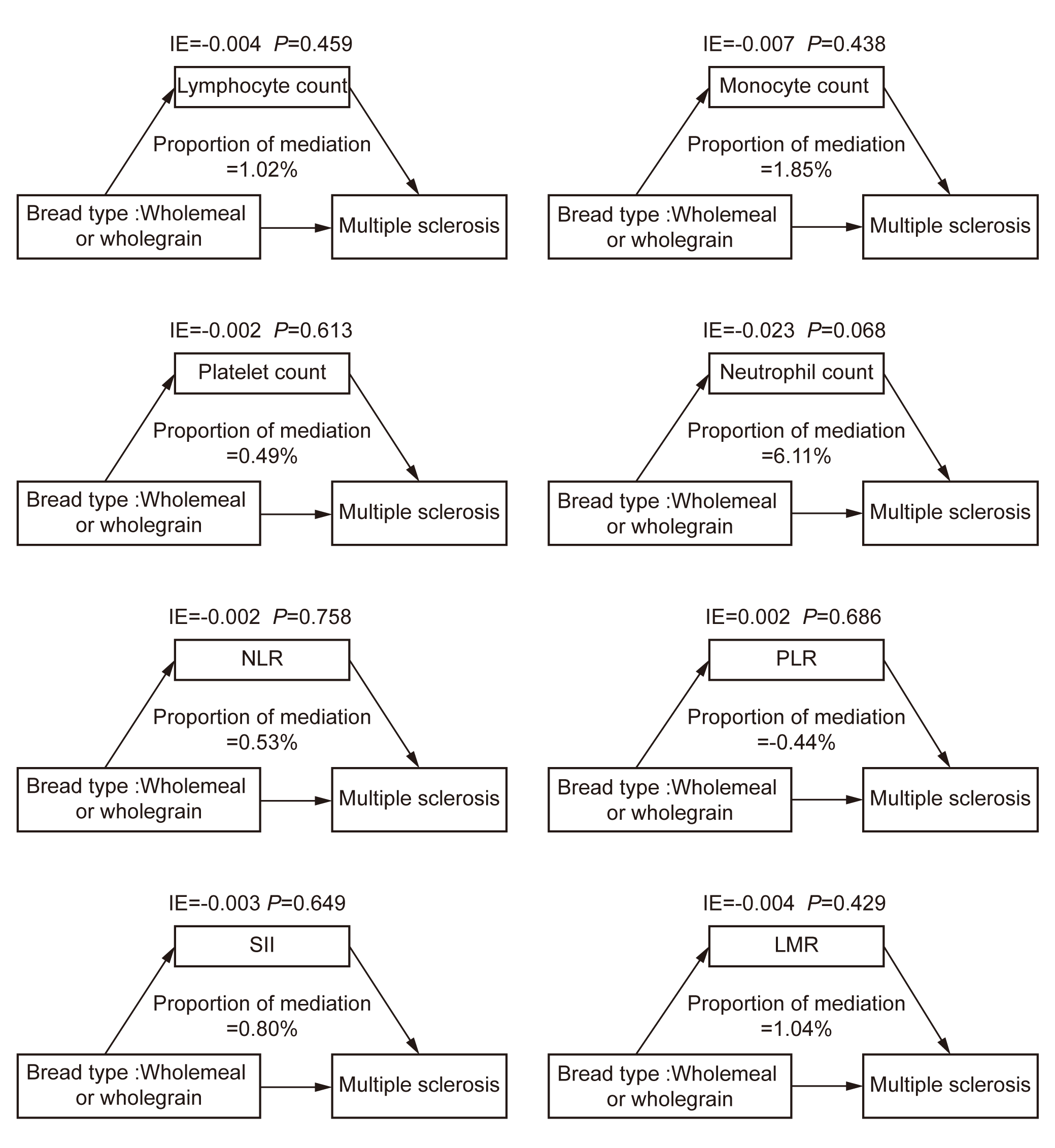

Supplement: Supplementary file 5 — Supporting file 5: mnfr70313‐sup‐0005‐figureS4.tif [file MNFR-69-e70313-s004.tif]
